# Encyclopedia of the Diseases of Children

**Published:** 1891-03

**Authors:** 


ENCLYCOPEDIA OF THE DISEASES OF CHILDREN.—BY J. M. KEATING, M. D., PUBLISHED BY J. P. LIPPINCOTT, PHILADELPHIA.

The third volume of this valuable work has reached the *Journal* office. The high merit of the preceding volumes, is wholly sustained in this. It comprises diseases of the digestive system, diseases of the genito-urinary organs, circulatory diseases, surgery and disease of bones and joints. We would direct especial attention to the first department. The greater frequency of intestinal over other disorders in children, renders the subject of great practical importance to the general practitioner and the able treatises herein found commend themselves to one seeking full and detailed information in this direction. The classification is simple, the order concise, the type most legible. This volume ranks second to none as a comprehensive and useful work in this branch of medicine.

